# Strategies to improve delivery of equitable and evidence-informed care for pregnant and birthing people with a substance use disorder in acute care settings: A scoping review protocol

**DOI:** 10.1371/journal.pone.0300183

**Published:** 2024-03-18

**Authors:** Carla King, Gregory Laynor, Jennifer McNeely, Adetayo Fawole, Matthew Lee, Mishka Terplan, Sugy Choi

**Affiliations:** 1 Department of Population Health, NYU Grossman School of Medicine, New York, New York, United States of America; 2 Health Sciences Library, NYU Grossman School of Medicine, New York, New York, United States of America; 3 Friends Research Institute, Baltimore, Maryland, United States of America; PLoS ONE, UNITED STATES

## Abstract

This protocol outlines a proposed scoping review to characterize evidence on implementation and quality improvement (QI) strategies that aim to improve equitable, evidence-informed care delivery for pregnant and birthing people with substance use disorder (SUD) in acute care. Untreated SUD during pregnancy is associated with an increased risk of overdose and severe maternal morbidity. Acute care settings are one important place to deliver equitable, evidence-informed clinical care. While clinical practice guidelines for substance use treatment and care of pregnant and birthing people with SUD exist, there are gaps in implementation. Our population of interest is pregnant and birthing people with SUD in an acute care setting. We will include US-based studies that describe or evaluate implementation or QI strategies, including experimental, observational, and descriptive studies published from 2016 to 2023. The proposed scoping review will be conducted in accordance with JBI methodology for scoping reviews and registered at OSF (registration number: BC4VZ). We will search MEDLINE (PubMed), CINAHL Complete (EBSCO), Scopus (Elsevier), and APA PsychInfo (Ovid) for published studies. Conference proceedings and Perinatal Quality Collaborative websites will be searched for grey literature. Two reviewers will independently screen then extract studies that meet inclusion criteria using a data extraction tool. The completion of this scoping review will help illuminate strengths and gaps in research and practice that aim to inform substance use treatment and care in acute care settings for pregnant and birthing people with SUD.

## Introduction

### Rationale

Untreated substance use disorder (SUD) during pregnancy and postpartum is associated with an increased risk of overdose death and severe maternal morbidity in the United States (US) [1–3]. From 2018 to 2021, there was a significant increase in overdose deaths during pregnancy and postpartum, matching a trend of increasing overdose deaths across the US [1]. Hospitalizations throughout pregnancy are 4-fold higher for people who have an opioid-affected delivery and are frequently associated with behavioral health-related diagnoses, including substance use [4].

While early interventions for untreated SUD in pregnancy are ideal, [5, 6] pregnant people with SUD may delay or avoid prenatal care, often because they are criminalized for their substance use [7–9]. For some pregnant people, acute care visits could be the first or only opportunity for providers to offer evidence-informed treatment and care [9–12]. Federal and state organizations, as well as expert groups, have developed and disseminated evidence-informed clinical practice guidelines and principles of care for pregnant and birthing people with SUD [10, 13–17]. There are, however, gaps in uptake of these guidelines in acute care settings, and punitive policies and practices around substance use in pregnancy persist [18].

During hospitalization, pregnant and birthing people with SUD continue to experience discrimination, judgment, and criminalization [7, 8, 19]. Pregnant and birthing people with SUD describe interactions with the health care system as “disparaging” and “unhelpful” [7]. During labor and delivery, some people with SUD report that they feel highly scrutinized by hospital staff who make judgements about their parenting skills, and do not trust that inpatient care teams can address their needs [8].

Medications for opioid use disorder (MOUD)–methadone and buprenorphine–are considered safe and effective first-line recommended treatment for pregnant people with opioid use disorder (OUD) [10, 13–16]. Studies, however, find that providers who treat pregnant people report inadequate knowledge about MOUD and discomfort with counseling on its use without additional training [9]. Even among pregnant people enrolled in treatment for OUD, most do not receive MOUD [20].

Persistent structural and institutional inequities, particularly at the intersections of racism, genderism, sexism, and classism, also contribute to gaps in the adoption and delivery of equitable and evidence-informed practices [21, 22]. Increases in unintentional drug overdoses are particularly concerning among American Indian/Native American and Black birthing people [3]. Yet, Black and Hispanic persons are less likely than White persons to receive MOUD during pregnancy [23, 24]. Birthing people of color are also more likely to be treated with inadequate doses of MOUD during labor and delivery [25]. These disparities in care are likely to persist without addressing the embedded structural and institutional racism within health care systems [22].

There is an urgent need to identify strategies that i) improve the uptake and delivery of evidence-informed, person-centered practices for pregnant and birthing people with SUD in acute care settings, and ii) counter structural inequities and racism throughout design and implementation [22]. Person-centered care, including support and advocacy from providers and peers during hospitalization, has the potential to promote medical trust and better outcomes for pregnant people with SUD [8, 10, 13]. Characterizing the implementation science and quality improvement (QI) strategies used by hospitals, communities, and providers to address this research to practice (or more specifically, clinical care guidelines to practice) gap in acute care settings is critical to inform scale-up and a future research agenda.

A comprehensive review of the evidence from both implementation science and QI in this area could help characterize existing strategies. A preliminary search of MEDLINE (PubMed), Google Scholar, OSF, and Prospero found no existing or planned reviews on this specific topic. Existing reviews on acute care interventions and transitions from hospital to the community focus on patients with SUD broadly, [26, 27] but do not specifically describe those that target pregnant or birthing people with gender-related social needs [28]. Our study extends beyond a previous review by Lim et al. [29], which primarily focused on evidence related to clinical practices in OUD and peri-delivery pain management. We broaden the scope to include a comprehensive examination of the implementation of existing guidelines for individuals hospitalized during pregnancy or at the time of giving birth. Other systematic reviews [30, 31] center around interventions that target the neonate during hospitalization (i.e. rooming-in), but not those that address substance-related care for the birthing person. Joshi et al. [32] reviewed women-centered models for OUD treatment in outpatient settings but did not examine uptake of clinical practices in the acute care setting or for SUD more broadly. Finally, a scoping review [33] characterized the evidence on implementation science theories and frameworks used in maternal care broadly but did not specifically examine implementation strategies for this population with SUD or in acute care settings.

To address this gap, we propose scoping methods given the breadth of this topic and a need to inform a more targeted systematic review of implementation and QI strategies in the future.

### Objective

The overall objective of this scoping review is to characterize the evidence on implementation and QI strategies (i.e., policies, tools, interventions, programs, education) that aim to improve SUD care delivery in acute care hospital settings (i.e. emergency department, medical inpatient, labor and delivery) for pregnant or birthing people with SUD. This includes strategies developed and implemented by multiple contexts (i.e., states, counties, hospital systems) that are intended to increase uptake and delivery of evidence-informed practices or clinical recommendations, or to address racialized or ethnicized disparities in care.

To accomplish this objective, we aim to 1) identify and characterize relevant strategies within acute care settings for pregnant and birthing people with SUD, including the intended purpose of the strategy and its consideration of racial and ethnic equity; 2) summarize measures used to evaluate implementation success or other clinical/service/process outcomes; and 3) summarize ongoing barriers or existing facilitators identified after implementation.

## Eligibility criteria

Table 1 summarizes the study’s eligibility criteria.

**Table 1:** Eligibility criteria.

	Inclusion	Exclusion
**Participants**	i) Pregnant or birthing people ii) Identified substance use disorder related to opioids, stimulants, cannabis, hallucinogens, inhalants, sedatives, hypnotics /anxiolytics	i) Identified tobacco or caffeine use disorder
**Concept**	Studies/reports that describe or evaluate: i) Strategies that aim to improve SUD treatment and care delivery for our target population in acute care settings	Studies/reports where the strategy targets: i) Screening or testing of pregnant people with suspected substance use ii) Neonate care only iii) Pre-implementation studies that examine the contextual barriers/facilitators or perceptions of strategies that have not yet been implemented
**Context**	i) Acute care settings (inpatient, labor/delivery, emergency services) ii) United States iii) Published from 2016 to 2023	i) Outpatient prenatal or postpartum programs ii) Outside of the United States iii) Published before 2016 or after 2023
**Type of study/ Source**	i) All original research including experimental/quasi-experimental, observational, implementation and descriptive studies ii) Quality improvement reports/sources iii) Qualitative studies describing perceptions of a strategy that has been implemented in an acute care setting	i) Commentaries ii) Dissertations iii) Review articles

### Participants

Our population of interest is hospitalized people with an identified SUD who are interacting with an acute care setting during antepartum or intrapartum/labor and delivery. SUD in our study is inclusive of alcohol and all other drug use disorders identified in The Diagnostic and Statistical Manual of Mental Disorders, Fifth Edition [34] (i.e., opioids, stimulants, cannabis, hallucinogens, inhalants, sedatives, hypnotics/anxiolytics), excluding tobacco and caffeine.

Of note, strategies used in acute care settings may be directed by or targeting populations other than our population of interest. We will include studies that aim to improve care for our population of interest, but where participants are not always pregnant or birthing people with SUD themselves. “Actors” (i.e., the person or group “who actually delivers the implementation strategy”) and “action targets” (i.e., “targets [that the strategy] attempts to impact”) could be hospital providers, hospital administrators, community organizations, or state organizations [35].

### Concept

We include studies or reports that describe or evaluate strategies used in acute care settings aimed to improve care delivery for our target population. This includes QI projects, implementation studies, and studies that describe participant perceptions of strategies after implementation.

We use the term “strategies” to encompass terminology from both implementation science and QI practice. “Strategies” in implementation science refer to implementation strategies, or “methods to enhance the adoption, implementation, sustainment, and scale-up of an innovation” [36]. The US Centers for Medicare and Medicaid Services [37] define QI as “the framework used to systematically improve care”. In QI, “strategies” may include “interventions or tools” such as Fishbone diagrams or process models [38]. While the methods and terminology differ, this review encompasses both QI and implementation science given their mutual aim to improve care outcomes for hospitalized patients and to recognize calls for integrating these practices to improve overall efficiency and effectiveness in improvement work [38–40].

Equity is a key consideration in both implementation science and QI, and experts recommend it be made explicit in design and implementation [41]. Given evident racialized and ethnicized inequities that lead to disparate treatment and care among groups in our population of interest, [23–25, 42–44] we will evaluate the inclusion of a racial or ethnic equity lens in the included studies. We will not, however, exclude studies based on the lack of a racial or ethnic equity lens.

This review focuses on pregnant and birthing people with an identified SUD, so will exclude studies where the strategy targets i) screening or testing of pregnant people with suspected substance use, and ii) neonate care only. We will also exclude pre-implementation studies that examine the contextual barriers/facilitators or perceptions of strategies that have not yet been implemented.

### Context

We will include studies that focus on SUD treatment and care in acute care settings (inpatient, labor/delivery, emergency services) in the US published between 2016 to 2023. The rationale for our study period is to capture recently implemented strategies, but also the period when relevant practice recommendations and their updates were published [10, 14, 15, 17, 45–48] as well as the launch of the National Network of Perinatal Quality Collaboratives in 2016. [49].

We exclude studies that focus on outpatient prenatal or postpartum programs (i.e., provide care outside of an acute care setting). Because the focus of this review is on uptake of US-based clinical care guidelines, we exclude studies from outside of the US.

### Type of study/source

We will include all original research including experimental/quasi-experimental, observational, implementation and descriptive studies. We will also include QI reports/sources and qualitative studies describing perceptions of a strategy that has been implemented in an acute care setting.

Commentaries and dissertations will be excluded. We will not include review articles but will screen the references of relevant review articles for any additional original research.

## Methods

This protocol follows the JBI Evidence Synthesis best practice guidance and reporting items for the development of scoping review protocols [50] and is reported in accordance with the Preferred Reporting Items for Systematic Review and Meta-analysis Protocols (PRISMA-P) guidelines, [51] as applicable. An information specialist/research librarian (GL) contributed to protocol development. The protocol is registered at OSF (registration number: BC4VZ).

The proposed scoping review will be conducted in accordance with JBI methodology for scoping reviews, [52] with guidance from Pollock et al. [53] and Chapman et al., [54] and be reported in accordance with Preferred Reporting Items for Systematic Reviews and Meta-Analyses extension for scoping reviews (PRISMA-ScR) [55]. Amendments to the protocol will be recorded and noted in the supplementary material of the scoping review.

### Search strategy

The search strategy will aim to locate both published and unpublished studies written in English. We will include studies published from 2016 to 2023. We will use EndNote 20.6 (Clarivate, PA, USA) [56] to manage the results of the search. The search will be conducted in January 2024 or later.

#### Published studies

We will search MEDLINE (PubMed), CINAHL Complete (EBSCO), Scopus (Elsevier), and APA PsychInfo (Ovid) for published studies. An initial search in MEDLINE (PubMed) identified keywords and structured vocabulary from relevant studies that guided the development of the core search strategy. We iteratively developed the components of our core search strategy in alignment with our eligibility criteria. The finalized core search strategy is presented in Table 2. Once finalized, the core search strategy was translated for CINAHL Complete (EBSCO), Scopus Advanced (Elsevier) and APA PsychInfo (Ovid) databases using the Polygot Translator, [57] and manual edits were made to search strings, as needed. We updated structural vocabulary, when available, to align with each database, while remaining consistent to our objective and core search strategy. Translated search strategies are included in Table A in S1 File.

**Table 2:** Core search strategy for MEDLINE (PubMed).

**Participants**	("Pregnant Women"[Mesh] OR "Delivery, Obstetric"[Mesh] OR Pregnancy[Mesh] OR pregnan*[Title/Abstract] OR birthing[Title/Abstract]) **AND** ("Substance-Related Disorders"[Mesh] OR Alcohol-Related Disorders[Mesh] OR Opioid-Related Disorders[Mesh] OR Drug Users[Mesh] OR "substance use"[Title/Abstract] OR "substance use disorder*"[Title/Abstract] OR "Drug use disorder"[Title/Abstract] OR "substance related disorder"[Title/Abstract] OR "substance abuse*"[Title/Abstract] OR "substance dependenc*"[Title/Abstract] OR "chemical dependenc*"[Title/Abstract] OR "drug addiction"[Title/Abstract] OR "opioid use disorder"[Title/Abstract] OR "opioid-related disorder*"[Title/Abstract] OR "alcohol-related disorder*"[Title/Abstract] OR "cocaine-related disorder*"[Title/Abstract] OR "alcohol use disorder"[Title/Abstract] OR "alcohol abuse"[Title/Abstract] OR "alcohol use"[Title/Abstract] OR "cannabis abuse"[Title/Abstract] OR "cannabis use disorder"[Title/Abstract] OR "cannabis use"[Title/Abstract] OR "marijuana abuse"[Title/Abstract] OR "marijuana use"[Title/Abstract] OR "inhalant abuse"[Title/Abstract] OR "inhalant use disorder"[Title/Abstract] OR "inhalant use"[Title/Abstract] OR "*amphetamine abuse"[Title/Abstract] OR "*amphetamine use disorder"[Title/Abstract] OR "*amphetamine use"[Title/Abstract] OR "stimulant abuse"[Title/Abstract] OR "stimulant use"[Title/Abstract] OR "opioid abuse"[Title/Abstract] OR "opioid use"[Title/Abstract] OR "cocaine abuse"[Title/Abstract] OR "cocaine use"[Title/Abstract] OR "polysubstance abuse"[Title/Abstract] OR "polysubstance use"[Title/Abstract] OR "overdose"[Title/Abstract] OR "drug overdose"[Title/Abstract] OR "SUD"[Title/Abstract] OR "OUD"[Title/Abstract] OR "AUD"[Title/Abstract] OR "SUDs"[Title/Abstract] OR "OUDs"[Title/Abstract] OR "AUDs"[Title/Abstract] OR "people who use drugs"[Title/Abstract] OR "drug user*"[Title/Abstract])
**Concept**	"Quality Improvement"[Mesh] OR "Implementation Science"[Mesh] OR "Evidence-Based Practice"[Mesh] OR "quality improvement"[Title/Abstract] OR "implementation"[Title/Abstract] OR "evidence-based practice*"[Title/Abstract] OR "project*"[Title/Abstract] OR "service*"[Title/Abstract] OR "practice*"[Title/Abstract
**Context**	("Delivery, Obstetric"[Mesh] OR "Nursing"[Mesh] OR "Hospitals"[Mesh] OR "Emergency Service, Hospital"[Mesh] OR "Labor, Obstetric"[Mesh] OR "Addiction Medicine"[Mesh] OR "acute care"[Title/Abstract] OR "labor/delivery"[Title/Abstract] OR "labor and delivery"[Title/Abstract] OR "labour/delivery"[Title/Abstract] OR "labour and delivery"[Title/Abstract] OR "emergency department"[Title/Abstract] OR "Hospital Emergency Services"[Title/Abstract] OR "hospital-based"[Title/Abstract] OR nurse[Title/Abstract] OR nurses[Title/Abstract] OR nursing[Title/Abstract] OR "obstetric delivery"[Title/Abstract] OR "addiction medicine"[Title/Abstract] OR "hospital*"[Title/Abstract]) **AND** ("2016"[Date—Publication]: "2023"[Date—Publication])

#### Unpublished studies/sources

Grey literature searches will be adapted from our core search strategy. We will search archived conference proceedings from three annual meetings from 2016 to 2023: Annual Conference on the Science and Dissemination and Implementation in Health [58], Association for Multidisciplinary Education and Research in Substance use and Addiction (AMERSA) [59], and Society for Maternal-Fetal Medicine [60]. Conference proceedings will be searched using key words such as “pregnancy”, “substance use”, “acute care”, “labor/delivery”, and “project”, consistent with our eligibility criteria and core search strategy. Final search terms will be documented in accordance with PRISMA-S [61].

Additionally, we will search State Perinatal Quality Collaborative (PQC) websites for active QI initiatives that match our eligibility criteria. PQCs provide infrastructure to partnered hospitals and health care providers, and support hospital-based obstetrics QI projects [49]. Using an online database from the National Network of Perinatal Quality Collaboratives, [62] we will search PQC websites that list “substance use disorder among pregnant people (including but not limited to opioids)” as a key initiative at the time of search.

We will additionally search websites of PQCs named in any published studies that meet our inclusion criteria to find any past unpublished reports. We will also include any relevant published articles or unpublished reports that are found on PQC websites during the search. The reference list of all included published and unpublished studies will be screened for additional relevant studies.

### Evidence selection

All published studies will be collated and uploaded into Covidence [63] and duplicates removed. CK and AF will complete a pilot test of the inclusion/exclusion criteria on a randomly selected subset of 50–100 titles and abstracts. After completing a pilot test, remaining titles and abstracts will be screened by the two reviewers independently using the established inclusion/exclusion criteria. CK and AF will then independently screen the full text of selected citations against the inclusion/exclusion criteria. Reviewers will have regular meetings to ensure interrater reliability, with a goal of Cohen’s kappa >.80 [64]. Any disagreements that arise between the reviewers at each stage of the screening process will be resolved through discussion, or by a 3^rd^ independent reviewer if consensus cannot be reached. We will record and report the results of the search, including reasons for exclusion at the full text screen, in accordance with PRISMA-ScR [55].

Unpublished studies/sources that meet inclusion criteria will be added as a citation to EndNote 20.6 (Clarivate, PA, USA) [56] and imported to Covidence [63] for data extraction.

### Data extraction

Data will be extracted by CK and AF using a data extraction tool developed by the review team. The proposed tool is included in Table B in S1 File. In preparation for this protocol, the proposed draft extraction template was pre-piloted by one reviewer (CK) on three studies that met inclusion criteria. Reports or summaries from PQC websites will be extracted using the same data extraction form. Where there are more than one study/report on the same project, we will combine them and complete one data extraction form.

The draft data extraction template will be modified and revised as necessary during the process of extracting data from each included evidence source. Modifications will be detailed in the scoping review. Any disagreements that arise between the reviewers during extraction will be resolved through discussion, or by a 3^rd^ independent reviewer if consensus cannot be reached.

We organized data extraction to align with our eligibility criteria and study aims. The “Participants” section includes information on the strategy’s actor and action target [35]. Study methodology, as well as theories, models or frameworks guiding design will be extracted as the “concept”. Finally, to describe the context, we will extract the study setting, including the region/state, population density, and hospital department (i.e., emergency department, inpatient, labor/delivery).

To characterize the strategies (Aim 1), we will extract: the gap, problem, or practice guideline that the strategy is addressing (i.e., provider bias, naloxone distribution, MOUD); the strategy; and any data on the use of a racial or ethnic equity lens. Identified strategies will be described and classified according to Leeman et al.’s [65] 5 classes of implementation strategies: 1) Dissemination strategies; 2) Implementation process strategies; 3) Integration strategies, 4) Capacity-building strategies, and 5) Scale-up strategies. Reviewers will be provided with a guidance document that summarizes Leeman et al.’s [65] definitions and provides examples. We chose this classification system because it uses terminology and examples that align with both implementation science and QI and is useful for synthesizing findings [65]. Strategies will be further described (i.e., actor, action target, frequency) using guidance from Proctor et al. [35].

To assess use of a racial or ethnic equity lens, we will extract *explicit* or *implicit* mentions of racial equity in the study. Considering Shelton et al.’s [22] recommendations for addressing structural racism in implementation science, we define *explicit* as describing or naming “racial equity”, “structural racism”, “structural competency” in the study and *implicit* as acknowledging that racialized disparities exist, but not using equity-specific language.

Outcome measures (Aim 2) related to implementation or processes will be extracted and grouped according to Proctor et al.’s [66]. Taxonomy of Implementation Outcomes, as applicable. We will consider the level of measurement (i.e., community, hospital, provider, or pregnant person with SUD) for each outcome. Because this is a scoping review, we will not evaluate study results, but will include a summary of results from the included studies.

Finally, we will extract any barriers or facilitators (Aim 3) reported as part of the study implementation (i.e., reported as results or in the discussion).

### Data analysis and presentation

To achieve our review objective and aims, we will synthesize data captured in the extraction tool using recommendations from Pollock et al. [53].

We will create a table to summarize the classified strategies (as defined by Leeman et al. [65]) represented in the studies. We will provide a summary description, using frequencies and percentages, of the strategies used, the gap/problem addressed by the strategies, the actors, and action targets. If there are a large number of studies (>30) with considerable geographic variation, we will use a map to visualize the region/state of studies and density of evidence. In a second table, we will display data on the studies that considered racial or ethnic equity by presenting the data on the explicit or implicit attention to equity in the study.

We will summarize the outcome measures used across studies in tabular form, including the level of analysis reported in the study.

Finally, we will map the identified barriers and facilitators to the updated Consolidated Framework for Implementation Research (CFIR) [67] to structure our analysis of the domains that influence implementation. A narrative summary will accompany tabular data and describe how the results relate to the review’s objective and aims.

Because this is a scoping review, we will not evaluate the strength of evidence.

## Discussion

This scoping review will help illuminate strengths and gaps in research and practice that aim to inform substance use treatment and care in acute care settings for pregnant and birthing people with SUD. While evidence-informed clinical guidelines exist to advise on treatment and care of pregnant and birthing people with SUD, uptake of recommendations and evidence-based practices can be slow [40] and racialized and ethnicized inequities persist [23–25, 42–44]. Broadly characterizing the literature of existing strategies may inform future systematic reviews that aim to establish a strategy’s effectiveness in relation to a desired change, or guide development of a comprehensive implementation study. Additionally, presenting the frequency and setting of ongoing barriers and facilitators, and bringing attention to racial and ethnic equity in some studies, may advise future studies/projects on how to address structural inequities from the outset.

### Limitations

Although we have developed a comprehensive and systematic search, it is likely that we have not captured all QI projects, especially given that some hospital systems may not publish their results. Searching PQC websites is one attempt to mitigate this limitation, however, we may still miss independently conducted QI projects. Implementation science is a relatively new field and databases either do not have related structured vocabulary or only recently adopted them (i.e., MEDLINE’s “Implementation Science” MeSH terms was introduced in 2019) [54]. To address this limitation, we included structured vocabulary when possible, and searched title/abstract key words such as “evidence-based intervention” “implementation”, and “project”. We may, however, still miss published implementation studies that were not indexed. Finally, we excluded studies of strategies that were implemented in hospitals outside of the US. While we recognize that may miss important strategies used in similar countries, we are targeting data from the US context to understand how strategies improve adoption of US-based clinical recommendations.

### Dissemination plans

We plan to publish the results of this review in a peer-reviewed journal and present the findings to relevant hospitals and provider champions through conferences and presentations.

## Supporting information

S1 File Supporting information. (DOCX)

S2 File PRISMA-P checklist. (DOCX)
