# Supplementary material for: Strategies to improve delivery of equitable and evidence-informed care for pregnant and birthing people with a substance use disorder in acute care settings: A scoping review protocol
Source: PLoS One. 2024 Mar 18;19(3):e0300183. doi: 10.1371/journal.pone.0300183 (PMC10947689; doi:10.1371/journal.pone.0300183)
Supplement: S1 File — (DOCX) [file pone.0300183.s001.docx]

**S1 File.**

**Table A. Search Strategies.**

| **Database** | **Search string** |
| --- | --- |
| MEDLINE (PubMed) | (((("Pregnant Women"[Mesh] OR "Delivery, Obstetric"[Mesh] OR Pregnancy[Mesh] OR pregnan*[Title/Abstract] OR birthing[Title/Abstract]) AND ("Substance-Related Disorders"[Mesh] OR Alcohol-Related Disorders[Mesh] OR Opioid-Related Disorders[Mesh] OR Drug Users[Mesh] OR "substance use"[Title/Abstract] OR "substance use disorder*"[Title/Abstract] OR "Drug use disorder"[Title/Abstract] OR "substance related disorder"[Title/Abstract] OR "substance abuse*"[Title/Abstract] OR "substance dependenc*"[Title/Abstract] OR "chemical dependenc*"[Title/Abstract] OR "drug addiction"[Title/Abstract] OR "opioid use disorder"[Title/Abstract] OR "opioid-related disorder*"[Title/Abstract] OR "alcohol-related disorder*"[Title/Abstract] OR "cocaine-related disorder*"[Title/Abstract] OR "alcohol use disorder"[Title/Abstract] OR "alcohol abuse"[Title/Abstract] OR "alcohol use"[Title/Abstract] OR "cannabis abuse"[Title/Abstract] OR "cannabis use disorder"[Title/Abstract] OR "cannabis use"[Title/Abstract] OR "marijuana abuse"[Title/Abstract] OR "marijuana use"[Title/Abstract] OR "inhalant abuse"[Title/Abstract] OR "inhalant use disorder"[Title/Abstract] OR "inhalant use"[Title/Abstract] OR "*amphetamine abuse"[Title/Abstract] OR "*amphetamine use disorder"[Title/Abstract] OR "*amphetamine use"[Title/Abstract] OR "stimulant abuse"[Title/Abstract] OR "stimulant use"[Title/Abstract] OR "opioid abuse"[Title/Abstract] OR "opioid use"[Title/Abstract] OR "cocaine abuse"[Title/Abstract] OR "cocaine use"[Title/Abstract] OR "polysubstance abuse"[Title/Abstract] OR "polysubstance use"[Title/Abstract] OR "overdose"[Title/Abstract] OR "drug overdose"[Title/Abstract] OR "SUD"[Title/Abstract] OR "OUD"[Title/Abstract] OR "AUD"[Title/Abstract] OR "SUDs"[Title/Abstract] OR "OUDs"[Title/Abstract] OR "AUDs"[Title/Abstract] OR "people who use drugs"[Title/Abstract] OR "drug user*"[Title/Abstract])) AND ("Quality Improvement"[Mesh] OR "Implementation Science"[Mesh] OR "Evidence-Based Practice"[Mesh] OR "quality improvement"[Title/Abstract] OR "implementation"[Title/Abstract] OR "evidence-based practice*"[Title/Abstract] OR "project*"[Title/Abstract] OR "service*"[Title/Abstract] OR "practice*"[Title/Abstract)) AND (("Delivery, Obstetric"[Mesh] OR "Nursing"[Mesh] OR "Hospitals"[Mesh] OR "Emergency Service, Hospital"[Mesh] OR "Labor, Obstetric"[Mesh] OR "Addiction Medicine"[Mesh] OR "acute care"[Title/Abstract] OR "labor/delivery"[Title/Abstract] OR "labor and delivery"[Title/Abstract] OR "labour/delivery"[Title/Abstract] OR "labour and delivery"[Title/Abstract] OR "emergency department"[Title/Abstract] OR "Hospital Emergency Services"[Title/Abstract] OR "hospital-based"[Title/Abstract] OR nurse[Title/Abstract] OR nurses[Title/Abstract] OR nursing[Title/Abstract] OR "obstetric delivery"[Title/Abstract] OR "addiction medicine"[Title/Abstract] OR "hospital*"[Title/Abstract]))) AND **(("2016"[Date - Publication] : "2023"[Date - Publication]))** |
| CINAHL Complete (EBSCO) | (((((TI hospital* OR AB hospital*) OR (TI "acute care" OR AB "acute care") OR (TI labor/delivery OR AB labor/delivery) OR (TI "labor and delivery" OR AB "labor and delivery") OR (TI labour/delivery OR AB labour/delivery) OR (TI "labour and delivery" OR AB "labour and delivery") OR (TI "emergency department" OR AB "emergency department") OR (TI "Hospital Emergency Services" OR AB "Hospital Emergency Services") OR (TI hospital-based OR AB hospital-based) OR (TI nurse OR AB nurse) OR (TI nurses OR AB nurses) OR (TI nursing OR AB nursing) OR (TI "obstetric delivery" OR AB "obstetric delivery") OR (TI "addiction medicine" OR AB "addiction medicine")) OR ((MH "Delivery, Obstetric+") OR (MH “Labor and Delivery Nurses”+) OR (MH Hospitals+) OR (MH "Emergency Medical Services+") OR (MH "Labor+") OR (MH "Addictions Nursing+") OR (MH "Nursing Staff, Hospital+") OR (MH "Obstetrics Service+") OR (MH "Social Work Service+"))) AND (((TI "substance use" OR AB "substance use") OR (TI "substance use disorder*" OR AB "substance use disorder*") OR (TI "Drug use disorder" OR AB "Drug use disorder") OR (TI "substance related disorder" OR AB "substance related disorder") OR (TI "substance abuse*" OR AB "substance abuse*") OR (TI "substance dependenc*" OR AB "substance dependenc*") OR (TI "chemical dependenc*" OR AB "chemical dependenc*") OR (TI "drug addiction" OR AB "drug addiction") OR (TI "opioid use disorder" OR AB "opioid use disorder") OR (TI "opioid-related disorder*" OR AB "opioid-related disorder*") OR (TI "alcohol-related disorder*" OR AB "alcohol-related disorder*") OR (TI "cocaine-related disorder*" OR AB "cocaine-related disorder*") OR (TI "alcohol use disorder" OR AB "alcohol use disorder") OR (TI "alcohol abuse" OR AB "alcohol abuse") OR (TI "alcohol use" OR AB "alcohol use") OR (TI "cannabis abuse" OR AB "cannabis abuse") OR (TI "cannabis use disorder" OR AB "cannabis use disorder") OR (TI "cannabis use" OR AB "cannabis use") OR (TI "marijuana abuse" OR AB "marijuana abuse") OR (TI "marijuana use" OR AB "marijuana use") OR (TI "inhalant abuse" OR AB "inhalant abuse") OR (TI "inhalant use disorder" OR AB "inhalant use disorder") OR (TI "inhalant use" OR AB "inhalant use") OR (TI "*amphetamine abuse" OR AB "*amphetamine abuse") OR (TI "*amphetamine use disorder" OR AB "*amphetamine use disorder") OR (TI "*amphetamine use" OR AB "*amphetamine use") OR (TI "stimulant abuse" OR AB "stimulant abuse") OR (TI "stimulant use" OR AB "stimulant use") OR (TI "opioid abuse" OR AB "opioid abuse") OR (TI "opioid use" OR AB "opioid use") OR (TI "cocaine abuse" OR AB "cocaine abuse") OR (TI "cocaine use" OR AB "cocaine use") OR (TI "polysubstance abuse" OR AB "polysubstance abuse") OR (TI "polysubstance use" OR AB "polysubstance use") OR (TI overdose OR AB overdose) OR (TI "drug overdose" OR AB "drug overdose") OR (TI SUD OR AB SUD) OR (TI OUD OR AB OUD) OR (TI AUD OR AB AUD) OR (TI SUDs OR AB SUDs) OR (TI OUDs OR AB OUDs) OR (TI AUDs OR AB AUDs) OR (TI "people who use drugs" OR AB "people who use drugs") OR (TI "drug user*" OR AB "drug user*")) OR ((MH "Substance Abuse, perinatal+") OR (MH "Substance Use Disorders+") OR (MH "Alcohol-Related Disorders+") OR (MH "Substance Abusers+")))) AND (((TI pregnan* OR AB pregnan*) OR (TI birthing OR AB birthing)) OR ((MH "Expectant Mothers+") OR (MH "Labor") OR (MH Pregnancy+)))) AND (((((MH "Quality Improvement+")) OR (MH "Implementation Science+")) OR (MH "Program Development+") OR (MH "Professional Practice, Evidence-based+")) OR ((TI "quality improvement" OR AB "quality improvement") OR (TI implementation OR AB implementation) OR (TI "evidence-based practice*" OR AB "evidence-based practice*") OR (TI "project*”OR AB "project*”) OR (TI "service*”OR AB "service*”) OR TI "practice*" OR AB "practice*")))  **Date filter: 2016 to 2023** |
| Scopus (Elsevier) | ( ( ( TITLE-ABS-KEY ( hospital* ) OR TITLE-ABS-KEY ( "acute care" ) OR TITLE-ABS-KEY ( labor/delivery ) OR TITLE-ABS-KEY ( "labor and delivery" ) OR TITLE-ABS-KEY ( labour/delivery ) OR TITLE-ABS-KEY ( "labour and delivery" ) OR TITLE-ABS-KEY ( "emergency department" ) OR TITLE-ABS-KEY ( "Hospital Emergency Services" ) OR TITLE-ABS-KEY ( hospital-based ) OR TITLE-ABS-KEY ( nurse ) OR TITLE-ABS-KEY ( nurses ) OR TITLE-ABS-KEY ( nursing ) OR TITLE-ABS-KEY ( "obstetric delivery" ) OR TITLE-ABS-KEY ( "addiction medicine" ) ) OR ( TITLE-ABS-KEY ( "Obstetric Delivery " ) OR TITLE-ABS-KEY ( nursing ) OR TITLE-ABS-KEY ( hospitals ) OR TITLE-ABS-KEY ( "hospital Emergency Service" ) OR TITLE-ABS-KEY ( "Obstetric Labor" ) OR TITLE-ABS-KEY ( "Addiction Medicine" ) OR TITLE-ABS-KEY ( "Hospital Nursing Service" ) OR TITLE-ABS-KEY ( " Hospital Obstetrics and Gynecology Department" ) OR TITLE-ABS-KEY ( " Hospital Social Work Department" ) ) ) ) AND ( ( TITLE-ABS-KEY ( "substance use" ) OR TITLE-ABS-KEY ( "substance use disorder*" ) OR TITLE-ABS-KEY ( "Drug use disorder" ) OR TITLE-ABS-KEY ( "substance related disorder" ) OR TITLE-ABS-KEY ( "substance abuse*" ) OR TITLE-ABS-KEY ( "substance dependenc*" ) OR TITLE-ABS-KEY ( "chemical dependenc*" ) OR TITLE-ABS-KEY ( "drug addiction" ) OR TITLE-ABS-KEY ( "opioid use disorder" ) OR TITLE-ABS-KEY ( "opioid-related disorder*" ) OR TITLE-ABS-KEY ( "alcohol-related disorder*" ) OR TITLE-ABS-KEY ( "cocaine-related disorder*" ) OR TITLE-ABS-KEY ( "alcohol use disorder" ) OR TITLE-ABS-KEY ( "alcohol abuse" ) OR TITLE-ABS-KEY ( "alcohol use" ) OR TITLE-ABS-KEY ( "cannabis abuse" ) OR TITLE-ABS-KEY ( "cannabis use disorder" ) OR TITLE-ABS-KEY ( "cannabis use" ) OR TITLE-ABS-KEY ( "marijuana abuse" ) OR TITLE-ABS-KEY ( "marijuana use" ) OR TITLE-ABS-KEY ( "inhalant abuse" ) OR TITLE-ABS-KEY ( "inhalant use disorder" ) OR TITLE-ABS-KEY ( "inhalant use" ) OR TITLE-ABS-KEY ( "*amphetamine abuse" ) OR TITLE-ABS-KEY ( "*amphetamine use disorder" ) OR TITLE-ABS-KEY ( "*amphetamine use" ) OR TITLE-ABS-KEY ( "stimulant abuse" ) OR TITLE-ABS-KEY ( "stimulant use" ) OR TITLE-ABS-KEY ( "opioid abuse" ) OR TITLE-ABS-KEY ( "opioid use" ) OR TITLE-ABS-KEY ( "cocaine abuse" ) OR TITLE-ABS-KEY ( "cocaine use" ) OR TITLE-ABS-KEY ( "polysubstance abuse" ) OR TITLE-ABS-KEY ( "polysubstance use" ) OR TITLE-ABS-KEY ( overdose ) OR TITLE-ABS-KEY ( "drug overdose" ) OR TITLE-ABS-KEY ( sud ) OR TITLE-ABS-KEY ( oud ) OR TITLE-ABS-KEY ( aud ) OR TITLE-ABS-KEY ( suds ) OR TITLE-ABS-KEY ( ouds ) OR TITLE-ABS-KEY ( auds ) OR TITLE-ABS-KEY ( "people who use drugs" ) OR TITLE-ABS-KEY ( "drug user*" ) ) OR ( TITLE-ABS-KEY ( "Substance-Related Disorders" ) OR TITLE-ABS-KEY ( "Alcohol-Related Disorders" ) OR TITLE-ABS-KEY ( "Opioid-Related Disorders" ) OR TITLE-ABS-KEY ( "Drug Users" ) ) ) AND ( ( ( TITLE-ABS-KEY ( pregnan* ) OR TITLE-ABS-KEY ( birthing ) ) OR ( TITLE-ABS-KEY ( "Pregnant Women" ) OR TITLE-ABS-KEY ( "Obstetric Delivery" ) OR TITLE-ABS-KEY ( pregnancy ) ) ) AND ( TITLE-ABS-KEY ( "Quality Improvement" ) OR TITLE-ABS-KEY ( "Implementation Science" ) OR TITLE-ABS-KEY ( "Evidence-Based Practice" ) ) OR ( TITLE-ABS-KEY ( "quality improvement" ) OR TITLE-ABS-KEY ( implementation ) OR TITLE-ABS-KEY ( "evidence-based practice*" ) OR TITLE-ABS-KEY ( "project*" ) OR TITLE-ABS-KEY ( "service*" ) OR TITLE-ABS-KEY ( "practice*" ) ) ) AND **PUBYEAR > 2015 AND PUBYEAR < 2024** |
| APA PsychInfo (Ovid) | ((“hospital*”.ti,ab. OR "acute care".ti,ab. OR “labor/delivery”.ti,ab. OR "labor and delivery".ti,ab. OR “labour/delivery”.ti,ab. OR "labour and delivery".ti,ab. OR "emergency department".ti,ab. OR "Hospital Emergency Services".ti,ab. OR “hospital-based”.ti,ab. OR “nurse”.ti,ab. OR “nurses”.ti,ab. OR “nursing”.ti,ab. OR "obstetric delivery".ti,ab. OR "addiction medicine".ti,ab.)  OR (exp “obstetrics”/ OR exp “Nursing”/ OR exp “Hospitals”/ OR exp “Emergency Services”/ OR exp “Labor (childbirth)”/ OR exp “Addiction Medicine”/ OR exp “Addiction treatment"/ OR exp "Nurses"/ OR exp "Behavioral Health Services"/)) AND (("substance use".ti,ab. OR "substance use disorder*".ti,ab. OR "Drug use disorder".ti,ab. OR "substance related disorder".ti,ab. OR "substance abuse*".ti,ab. OR "substance dependenc*".ti,ab. OR "chemical dependenc*".ti,ab. OR "drug addiction".ti,ab. OR "opioid use disorder".ti,ab. OR "opioid-related disorder*".ti,ab. OR "alcohol-related disorder*".ti,ab. OR "cocaine-related disorder*".ti,ab. OR "alcohol use disorder".ti,ab. OR "alcohol abuse".ti,ab. OR "alcohol use".ti,ab. OR "cannabis abuse".ti,ab. OR "cannabis use disorder".ti,ab. OR "cannabis use".ti,ab. OR "marijuana abuse".ti,ab. OR "marijuana use".ti,ab. OR "inhalant abuse".ti,ab. OR "inhalant use disorder".ti,ab. OR "inhalant use".ti,ab. OR "*amphetamine abuse".ti,ab. OR "*amphetamine use disorder".ti,ab. OR "*amphetamine use".ti,ab. OR "stimulant abuse".ti,ab. OR "stimulant use".ti,ab. OR "opioid abuse".ti,ab. OR "opioid use".ti,ab. OR "cocaine abuse".ti,ab. OR "cocaine use".ti,ab. OR "polysubstance abuse".ti,ab. OR "polysubstance use".ti,ab. OR overdose.ti,ab. OR "drug overdose".ti,ab. OR SUD.ti,ab. OR OUD.ti,ab. OR AUD.ti,ab. OR SUDs.ti,ab. OR OUDs.ti,ab. OR AUDs.ti,ab. OR "people who use drugs".ti,ab. OR "drug user*".ti,ab.) OR (exp " Substance Related and Addictive Disorders"/ OR exp " substance use disorder"/ OR exp "alcohol use disorder"/ OR exp "alcohol abuse”/ OR exp "Opioid use Disorder"/ OR exp "Drug usage"/ OR exp "Drug abuse"/))AND ((pregnan*.ti,ab. OR birthing.ti,ab.) OR (exp "expectant mothers"/ OR exp "birth"/ OR exp Pregnancy/))AND ((exp "Quality of care"/ OR exp "Program development"/ OR exp "Evidence based Practice"/) OR ("quality improvement".ti,ab. OR implementation.ti,ab. OR "evidence-based practice*".ti,ab. OR "project*”.ti,ab. OR "service*".ti,ab. OR "practice*.ti,ab”))  **Date filter: 2016 to 2023** |

**Table B. Draft Data Extraction Template.**

| **#** | **Extraction Item** | **Responses** | |
| --- | --- | --- | --- |
| **Extraction Details** | | | |
| 1 | Extractor initials | Free text | |
| 2 | Date of extraction | Free text | |
| **Citation Details** | | | |
| 3 | Authors | Free text | |
| 4 | Publication year | Free text | |
| 5 | Title (copy and paste) | Free text | |
| 6 | Abstract (copy and paste) | Free text | |
| **Participants** | | | |
| 7 | Who will enact the strategy?  (i.e., the actor) [1] | Select all that apply:  Patients  Hospital providers  Hospital administrators  Educators  Community  States  Perinatal Quality Collaborative  Other (please specify): | |
| 8 | Who is the target of the actions? (i.e., the action target) [1] | Select all that apply:  Patients  Hospital providers  Hospital administrators  Community  States  Students (Medical/Nursing)  Other (please specify): | |
| 9 | What class of substance use does the strategy address? | Select all that apply:  Any substance/”substance use disorder”  Opioids  Alcohol  Stimulants  Cannabis  Hallucinogens  Inhalants  Sedatives  Hypnotics/anxiolytics  Other (please specify): | |
| **Concept** | | | |
| 10 | Study type | Select one:  Descriptive (including program descriptions)  Quality improvement project  Observational (excluding quality improvement projects)  Experimental/Quasi-experimental/Pragmatic | |
| 11 | Methods | Select one:  Qualitative  Quantitative  Both  N/A (i.e., descriptive study without outcomes) | |
| 12 | Design | Select one:  Cross-sectional  Prospective  Retrospective | |
| 13 | Theories/Models/Frameworks guiding study design | Free text | |
| **Context** | | | |
| 14 | Country | United States  United States and other countries (specify other countries): | |
| 15 | Region/State | 1. Free text 2. Select all that apply:   Rural  Urban  Suburban  Other:  Not specified | |
| 16 | Target hospital setting of care delivery | Select all that apply:  Emergency Department  Inpatient  Labor/Delivery/NICU (specifically)  Overall hospital  Other: | |
| 17 | Study period  (i.e., period of data collection or implementation) | Free text | |
| **Aim 1 \| Identify and classify strategies** | | | |
| 18 | What is the gap/problem/practice guideline that the strategy is addressing? (i.e., provider bias; naloxone distribution) | Free text | |
| 19 | What strategies/approaches were used? | 1. Briefly describe the strategy (i.e., the actions, steps, or processes that needed to be enacted [1] (free text): 2. If applicable, describe dose of the strategy (i.e., duration, frequency) (free text): 3. Classify the strategy using Leeman et al. [2]:   1) Dissemination strategy  2) Implementation process strategy  3) Integration strategy  4) Capacity-building strategy  5) Scale-up strategy | |
| 20 | Does the study mention racial and/or ethnic equity? | 1. Yes/No 2. If yes, Is it explicit or implicit? (free text):   **explicit:** describes or names “racial equity”, “structural racism”, “structural competency”  **implicit:** acknowledges that racialized disparities exist, but not using equity-specific language   1. Copy/paste any specific definitions: 2. Where was it mentioned? (select all that apply):   Background/Introduction  Methods  Results  Discussion/Limitations  Other (please specify):   1. Was it operationalized in the study? Yes/No 2. Briefly describe (i.e., considered in study design, noted in discussion as future direction) (free text) | |
| **Aim 2 \| Summarize the outcome measures** | | | |
| 21 | Are implementation outcomes measured? (As defined by Proctor et al.[3]) | 1. Yes/No 2. If yes, select all that apply:   Acceptability  Appropriateness  Feasibility  Adoption  Fidelity  Cost  Penetration  Sustainability  Other/Not listed (please specify):   1. At what level were the outcomes measured?   Community  Hospital  Providers  Patients   1. What were the results? (copy and paste from the results section): | |
| 22 | Are other clinical/service/process outcomes measured?  (i.e., overdose, acute care utilization) | 1. Yes/No 2. If yes, describe (copy and paste from methods): 3. What were the results (copy and paste from results)? | |
| **Aim 3 \| Summarize barriers or facilitators identified after implementation** | | | |
| 23 | Reported barriers (specific to this study, in results or discussion sections) | | Free text |
| 24 | Reported facilitators (specific to this study, reported in results or discussion sections) | | Free text |
| **Other notes** | | | |
| 25 | Any additional notes | | Free text |
| 26 | Exemplar quotes (qualitative or mixed methods studies only) | | Free text |

**References**

1. Proctor EK, Powell BJ, McMillen JC. Implementation strategies: recommendations for specifying and reporting. Implement Sci. 2013;8:139. Epub 20131201. doi: 10.1186/1748-5908-8-139.

2. Leeman J, Birken SA, Powell BJ, Rohweder C, Shea CM. Beyond "implementation strategies": classifying the full range of strategies used in implementation science and practice. Implement Sci. 2017;12(1):125. Epub 20171103. doi: 10.1186/s13012-017-0657-x.

3. Proctor E, Silmere H, Raghavan R, Hovmand P, Aarons G, Bunger A, et al. Outcomes for implementation research: conceptual distinctions, measurement challenges, and research agenda. Adm Policy Ment Health. 2011;38(2):65-76. doi: 10.1007/s10488-010-0319-7.
